# Metal–Organic Framework Materials for Production and Distribution of Ammonia

**DOI:** 10.1021/jacs.2c06216

**Published:** 2023-01-23

**Authors:** Xue Han, Sihai Yang, Martin Schröder

**Affiliations:** Department of Chemistry, University of Manchester, Manchester M13 9PL, U.K.

## Abstract

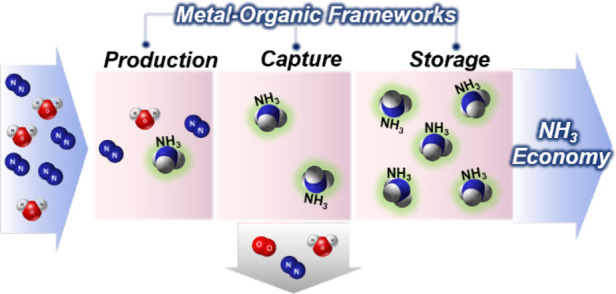

The efficient production of ammonia (NH_3_) from dinitrogen (N_2_) and water (H_2_O) using renewable energy is an important step on the roadmap to the ammonia economy. The productivity of this conversion hinges on the design and development of new active catalysts. In the wide scope of materials that have been examined as catalysts for the photo- and electro-driven reduction of N_2_ to NH_3_, functional metal–organic framework (MOF) catalysts exhibit unique properties and appealing features. By elucidating their structural and spectroscopic properties and linking this to the observed activity of MOF-based catalysts, valuable information can be gathered to inspire new generations of advanced catalysts to produce green NH_3_. NH_3_ is also a surrogate for the hydrogen (H_2_) economy, and the potential application of MOFs for the practical and effective capture, safe storage, and transport of NH_3_ is also discussed. This Perspective analyzes the contribution that MOFs can make toward the ammonia economy.

## 1 Introduction

Ammonia (NH_3_) is produced biologically under ambient conditions through the action of the enzyme, *Nitrogenase*, which is found only in a select group of microorganisms.^1,2^ Industrial production of NH_3_ was first developed by Haber and scaled-up by Bosch (Haber–Bosch process) more than 100 years ago.^3^ This process is currently estimated to turn over 200 million tonnes of NH_3_ per year, and between 75% and 90% of the NH_3_ produced is used as fertilizers for global food production. However, the Haber–Bosch process operates generally at high temperature (573–773 K) and high pressure (100–200 bar) in order to break the highly stable N≡N bond (dissociation energy of 941 kJ mol^–1^), and is thus regarded as one of the most energy-intensive industrial processes.

Using NH_3_ as a fuel is less common compared with its application in the agricultural sector, but it also has a long history dating back to World War II when NH_3_–coal gas hybrid motors were developed to maintain public transportation during diesel shortages.^4^ However, the development of human society is mainly sustained by carbon-based fuels and feedstocks due to abundant natural reserves, and with rapid global population growth and ever-increasing demand for energy, consumption of fossil fuels has led to increasing levels of CO_2_ in the environment with concomitant global warming.

The current unsustainability of the “Carbon Economy” and the environmental impacts of excess CO_2_ emissions have ignited searches for alternative fuels.^5^ Hydrogen (H_2_) has been widely recognized as a promising clean fuel with many new technologies being developed to build the “Hydrogen Economy”. But, many hurdles are still to be overcome including the storage and transport of H_2_ economically at high capacity.^6,7^ As a result, NH_3_ (containing no carbon) has regained the attention of many researchers and global organizations as an alternative to fossil fuels.^8^ The energy density of NH_3_ by volume is nearly double that of liquid H_2_, and NH_3_ is readily stored and transported since it can be liquefied by pressurizing to ∼10 bar at room temperature or by cooling to −33 °C at atmospheric pressure. NH_3_ is also considered clean in the sense that potential products of conversion are benign H_2_O and N_2_. However, unlike naturally occurring fossil fuels, NH_3_ is only produced to large-scale by industrial processes via conversion of N_2_ and H_2_ into NH_3_.

The Haber–Bosch process is the cornerstone of the modern NH_3_ industry, and it has been optimized continuously over the past century. Overall, the production of NH_3_ consumes about 2% of the world’s energy, and thus, if NH_3_ were to be used at large scale as a sustained medium to store and distribute energy, moving away from current Haber–Bosch technologies would be required. The production of NH_3_ from renewable H_2_ from water and N_2_ from the atmosphere is an attractive option. An ideal solution would be to drive this conversion with sunlight or renewable electricity, thus storing these intermittent sustainable energies in the form of chemical energy that can be extracted as required. Compared with the established infrastructure supporting carbon fossil fuels and rapidly emerging technologies based on batteries and H_2_-driven fuel cells, the application of NH_3_ in the energy sector is still in its infancy. New concepts, materials, and technologies are required, from the atomic-level fundamental design of catalysts to the development of associated engineering infrastructure.

**Scheme 1:**
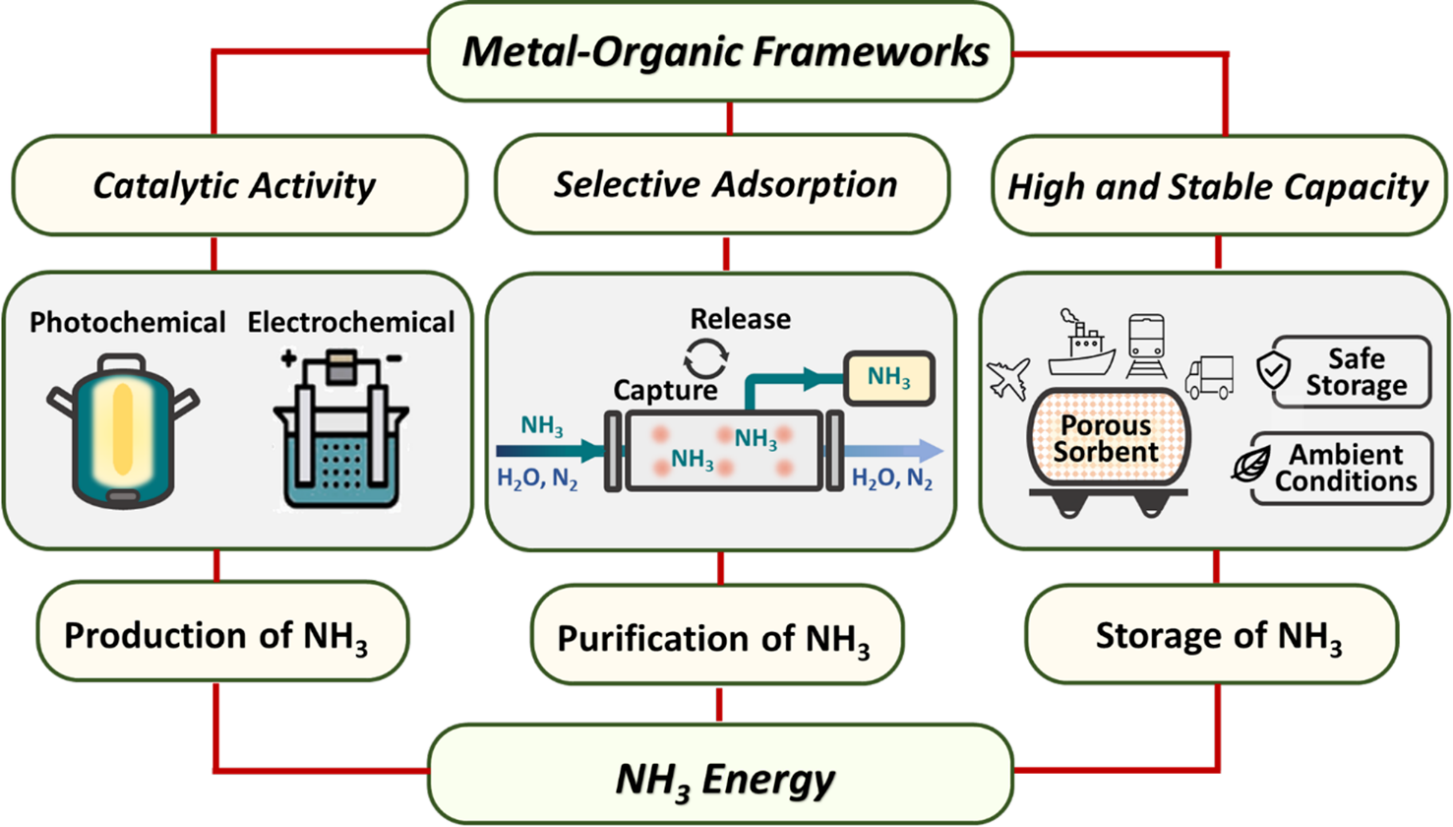
Schematic Showing the Potential Contribution of MOFs toward the Development of NH_3_ Energy

In this Perspective, we focus on porous metal–organic frameworks (MOFs) as emerging advanced functional materials and catalysts, particularly on (i) their application to the catalytic production of NH_3_*via* the photochemical and electrochemical nitrogen reduction reaction (NRR), (ii) the efficient capture of trace NH_3_ from gas mixtures, and (iii) safe storage of NH_3_ for its distribution and end-use (Scheme 1). As versatile functional materials, MOFs can exhibit ultra-high surface area (over 7000 m^2^ g^–1^) and porosity with highly dispersed but ordered metal/organic sites. Combining their great synthetic tunability and structural control, MOFs have shown great advantages as catalysts and as precursor catalysts in a large range of reactions. These works have been reviewed extensively.^9,10^ Depending on the nature of the catalytic transformation and the reaction conditions, diverse structural features of MOFs can be exploited. For example, in photo-/electrocatalytic reactions, their abilities to adsorb the substrate preferentially, to aid the transport of photoinduced electrons *via* metal-to-ligand or ligand-to-metal charge transfer, to extend the lifetime of the photoactivated species, and to afford rich defect sites for binding reactants are particularly appealing in achieving high productivity and selectivity for desired products.^11−13^ Meanwhile, the tailored design of MOFs can afford ideal pore environments to enable high adsorption capacities and efficient packing of NH_3_ molecules, coupled to exceptional adsorption selectivity and density of storage for applications in the transport sector. However, the corrosive and caustic nature of NH_3_ requires the storage material to be highly stable and robust to this reactive substrate, which can be highly challenging.

## 2 MOF-Assisted Photochemical Synthesis of NH_3_*via* Reduction of N_2_

Early examples of photocatalytic reduction of N_2_ under ambient conditions date back to the 1970s, where TiO_2_ was investigated as the photocatalyst.^14^ Since this pioneering work, numerous semiconductor-based materials have been investigated as catalysts, including metal oxides, metal sulfides, and metal-free semiconductors, and the active sites of these catalysts are mainly based on Fe, Ti, Ni, Mo, and the oxygen vacancies on the surface.^15^ This parallels and complements an established field of research on catalytic N_2_ reduction to hydrazine, NH_3_, and various intermediates, with associated mechanistic investigations, using homogeneous catalysts such as molybdenum and iron complexes.^16^ This area has been reviewed elsewhere^17^ and is beyond the scope of this Perspective.

**Figure 1:**
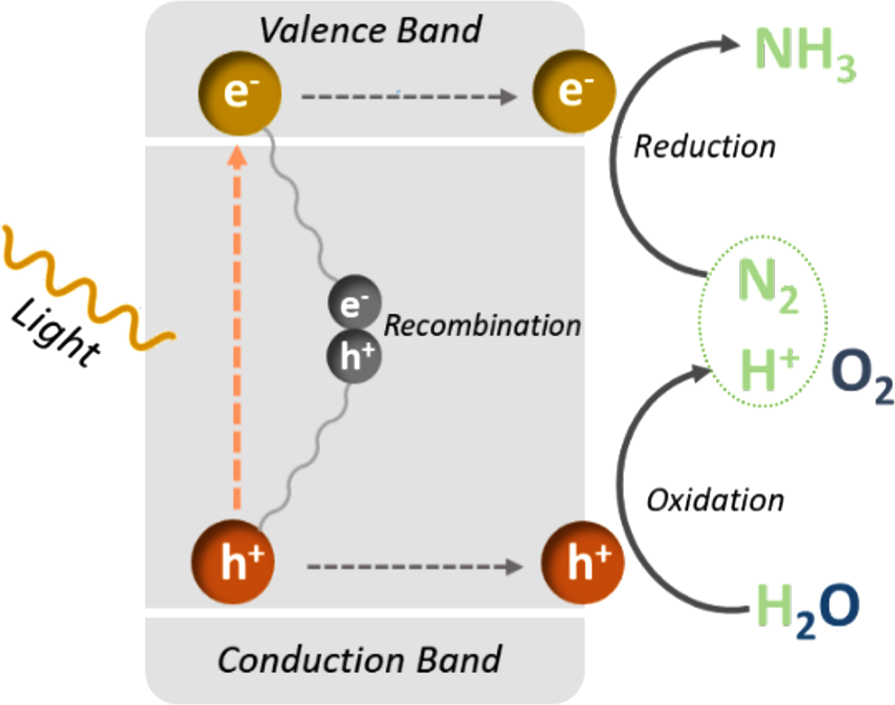
Schematic showing the general mechanism of photochemical reduction of N_2_ in the presence of H_2_O.

The general mechanism for photocatalytic conversion of N_2_ and H_2_O to NH_3_ usually involves light absorption, charge separation, binding of substrate, and its catalytic conversion (Figure 1), with detailed discussion being covered in recent review articles.^18^ MOFs could potentially provide an advantageous scaffold for assembling these components in 3D space in fixed positions, and the porous nature of the framework and extended metal–ligand coordination network can facilitate the mass transport of reactants and charge transfer, respectively. For these reasons, MOFs have been extensively explored as photocatalysts for CO_2_ reduction and water splitting,^11,13^ and this work has also inspired recent exploration of their applications in N_2_ reduction.

### 2.1 MOFs as Photocatalysts for NRR

**Figure 2:**
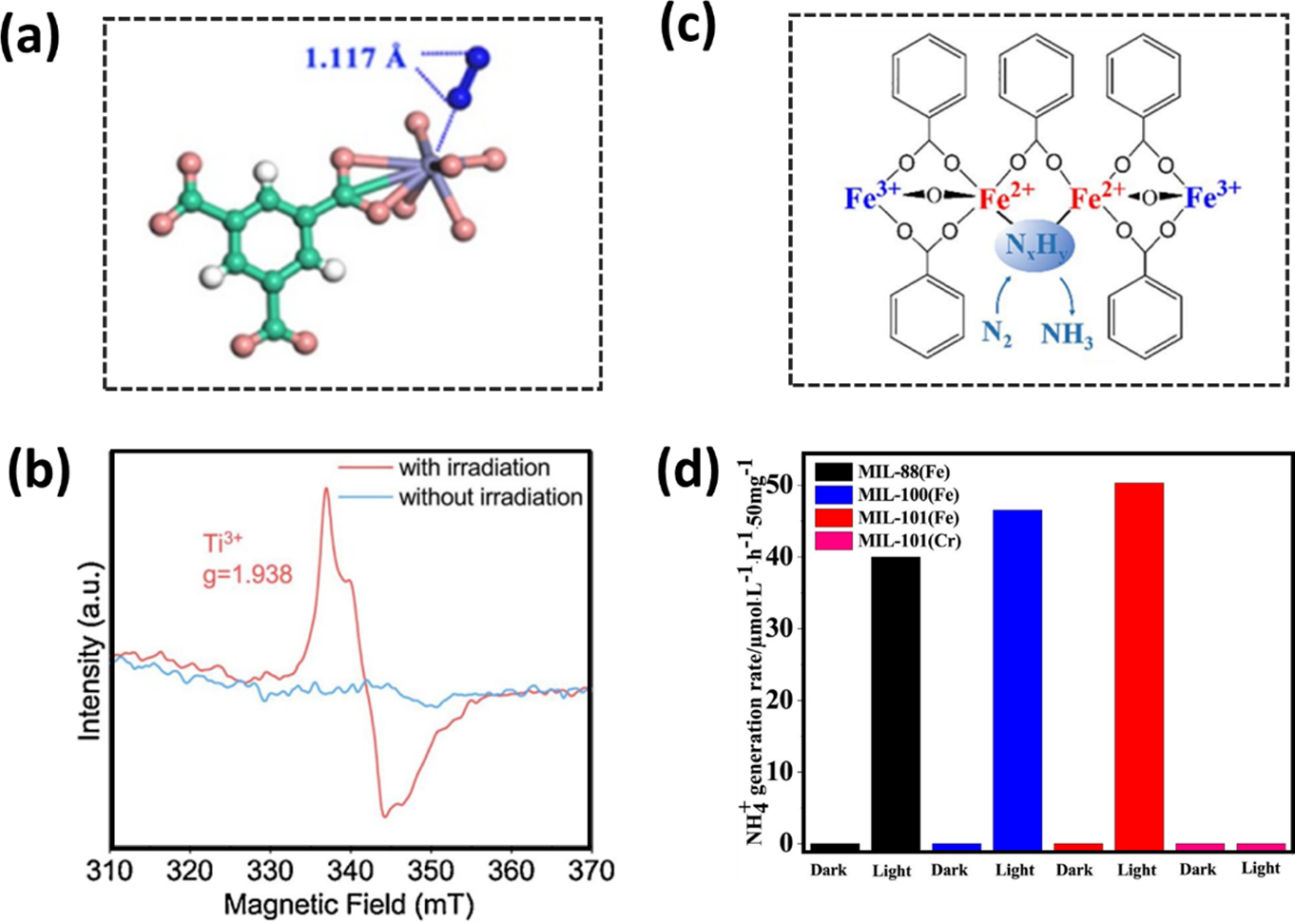
(a) Optimal structure of adsorption geometry of N_2_ on Ce^3+^-site (C, green; O, red; H, white; N, blue; Ce, purple). Reproduced with permission from ref (19). Copyright ACS 2019. (b) Low-temperature electron paramagnetic resonance (EPR) spectra of the as-prepared NH_2_-MIL-125(Ti) before and after light irradiation. Reproduced with permission from ref (22). Copyright Elsevier 2020. (c) View of the structure of MIL-53(Fe^2+^/Fe^3+^) photocatalyst. Reproduced with permission from ref (23). Copyright Elsevier 2020. (d) Photocatalytic nitrogen fixation activities of as-synthesized MIL-88, MIL-100, and MIL-101. Reproduced with permission from ref (25). Copyright Elsevier 2020.

A crucial first step to reduce N_2_ is to activate the strong N≡N bonds *via* interaction with an active site. Metal centers with partially occupied *d* (sometimes *f*) orbitals are of appropriate energy and symmetry for π back-bonding to N_2_ molecules. Cerium with [Xe]4f^2^6s^2^ electron configuration shows a valence-swing between 4f^1^ Ce(III) and 4f^0^ Ce(IV) oxidation states, and the Ce-based MOF-76(Ce) bearing coordinatively unsaturated Ce(III)/(IV) sites has been synthesized with benzene-1,3,5-tricarboxylic acid (trimesic acid) and investigated for photocatalytic N_2_ reduction.^19^ The empty 4f orbital of Ce(IV) sites can accept electrons from the σ orbital of N_2_, and the reduced Ce(III) back-donates electrons to the π* antibonding orbital of N_2_. Such π back-donation weakens and lengthens the N≡N triple bond to 1.117 Å, intermediate between the triple bond length (1.078 Å) of free N_2_ and the double bond length (1.201 Å) of diazene, HN=NH (Figure 2a). Experimental data and theoretical calculations confirmed that the trimesic acid linker is responsible for light absorption and electron excitation, with electrons transferred to Ce(IV) *via* ligand-to-metal charge transfer (LMCT) to form Ce(III) centers. MOF-76(Ce) exhibits a rate of formation of NH_3_ of 34 μmol g^–1^ h^–1^ under the reported conditions. This is a higher activity than that observed for CeO_2_ (4.5 μmol g^–1^ h^–1^), suggests that the environment of Ce(IV) and Ce(III) sites within the MOF can be of advantage for activation and reduction of N_2_. The Brunauer–Emmett–Teller (BET) surface area of MOF-76(Ce) is low at 13.9 m^2^ g^–1^ with only 10–15% of the Ce ions in the structure available for binding gas molecules. This would suggest that there is ample scope for improvements in catalytic activity by increasing the porosity and the proportion of active sites in the pore interior.

Inspired by the observed photocatalytic conversion of N_2_ to NH_3_ using TiO_2_ doped with Ti(III) formed by surface oxygen vacancies^20^ and by an early example of Ti^3+^-exchanged zeolites,^21^ several Ti-containing MOFs have been explored for N_2_ reduction, including CH_3_-MIL-125(Ti), OH-MIL-125(Ti), and NH_2_-MIL-125(Ti), which were synthesized by substitution of ligands in the UV-active MIL-125(Ti).^22^ Amine-functionalized NH_2_-MIL-125(Ti) shows the highest rate of formation for NH_3_ (12.3 μmol g^–1^ h^–1^) of the tested MOFs. It was confirmed that upon visible light irradiation, a long-lived charge-separated excited state was formed *via* LMCT to Ti(IV) to form Ti(III), with water acting as an electron donor (Figure 2b). N_2_ was captured by defect sites within the {Ti_8_} clusters leading to reduction of N_2_ molecules by Ti(III) sites to form NH_3_ and regeneration of Ti(IV) sites. Although the rate of formation of NH_3_ over NH_2_-MIL-125(Ti) catalyst is relatively low, it demonstrated that the catalytic activity for NRR can be tailored effectively by functionalization of the organic bridging ligands.

The role of metal sites in photocatalysts for NRR has been investigated in a series of MIL-53(Fe)-type materials incorporating mixed-valence Fe(II)/(III) clusters.^23^ In contrast to NH_2_-MIL-125(Ti) where LMCT was involved, the visible-light response in MIL-53(Fe) originates from the direct photoexcitation of Fe-oxo clusters. Compared with non-active MIL-53(Fe^3+^), the mixed-valence MIL-53(Fe^2+^:Fe^3+^ = 1.06:1) system displays a rate of formation for NH_3_ of 306 μmol h^–1^ g^–1^ (Figure 2c). Mixed-valence metals or mixed-metals clusters are accessible^24^ and may offer significant potential to mimic *Nitrogenase*-like structure and activity. Although MIL-53(Fe^3+^) is inactive, several other Fe(III)-based materials, including MIL-88(Fe), MIL-100(Fe), and MIL-101(Fe), all exhibit moderate photochemical activity for N_2_ reduction with rates of formation for NH_3_ of 80.0, 93.1, and 101 μmol h^–1^ g^–1^, respectively (Figure 2d).^25^ Interestingly, while MIL-101(Fe) exhibited the highest activity, its Cr analogue, MIL-101(Cr), was found to be inactive for photochemical NNR. This marked difference in catalytic activity is likely caused by a combination of factors, including primarily the electron configuration of the metal center (d^5^ for Fe^3+^ vs d^3^ for Cr^3+^) as well as the charge-transfer properties, porosity, and surface area of the samples.

**Figure 3:**
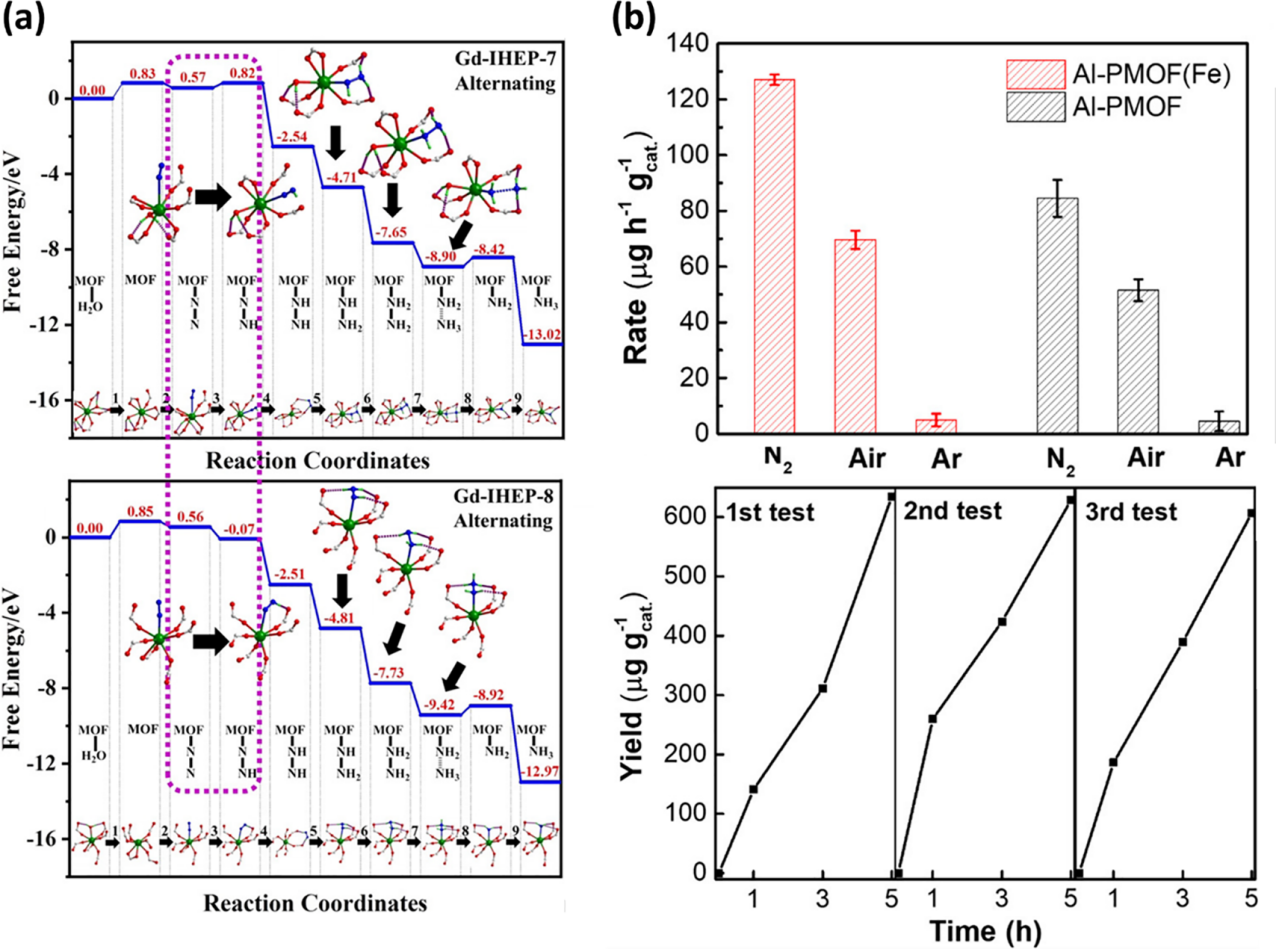
(a) Calculated energy diagrams for pathways for N_2_ reduction on Gd-IHEP-7 and Gd-IHEP-8 (Gd, green; C, gray; O, red; N, blue; H, light green). Reproduced with permission from ref (26). Copyright Wiley-VCH 2020. (b) Photocatalytic NRR rates for Al-PMOF and Al-PMOF(Fe) under N_2_, air, and Ar atmosphere. Reproduced with permission from ref (28). Copyright ACS 2021.

The catalytic performance of photocatalysts based upon MOFs can also be tuned *via* manipulation of the coordination environment of the metal center, as demonstrated by the viologen-based layered material Gd-IHEP-7. Upon heating in air, Gd-IHEP-7 undergoes a single-crystal-to-single-crystal transformation to generate a 3D material Gd-IHEP-8.^26^ Both MOFs exhibit excellent air and water stability and show a wide spectral absorption in the range 200–2500 nm to form stable radicals. Gd-IHEP-8 showed a higher rate of formation for NH_3_ than Gd-IHEP-7, 220 and 128 μmol g^–1^ h^–1^, respectively. Unlike the 9-coordinated Gd(III) center in Gd-IHEP-7, the 8-coordinated Gd(III) center in Gd-IHEP-8 can provide additional binding sites for reaction intermediates, thereby lowering the free energy of reaction (Figure 3a). In addition to applying heating to induce crystal phase transitions, light irradiation was also found to be effective in generating coordinatively unsaturated metal sites by formation of linker or metal cluster defects in the {Zr_6_}-based UiO-66.^27^ Photoactivated UiO-66 possessing rich linker defect sites exhibits a rate of formation for NH_3_ of 196 μmol g^–1^ h^–1^ in air under ultraviolet–visible (UV–vis) irradiation, higher than for as-synthesized UiO-66 (126 μmol g^–1^ h^–1^).

In addition to the metal nodes of the MOF scaffold, other active metal centers can be incorporated into the framework. This is exemplified by the porphyrin-based Al-PMOF.^28^ Upon insertion of an Fe(III) center to each porphyrin ring to form Al-PMOF(Fe), the rate of formation of NH_3_ is increased to 7.5 μmol g^–1^ h^–1^ compared to Al-PMOF (5.0 μmol g^–1^ h^–1^) (Figure 3b). DFT calculations reveals that N_2_ binds preferentially to the Fe(III) sites between two layers of PMOF, which also serve as the trap of the photogenerated electrons to inhibit the electron–hole recombination.

The performance of MOF photocatalysts for NRR is largely determined by their (i) light-absorbing and electron-transferring properties, (ii) ability to interact with and activate N_2_, and (iii) interaction with reaction intermediates to lower their free energy. The above cases exemplify that, by functionalizing the ligands and regulating the coordination environment of metal centers and clusters, the properties of MOF-based photocatalysts for N_2_ reduction can be tuned *via* property-led design of functional materials.

### 2.2 MOFs in Composite Photocatalysts for NRR

**Figure 4:**
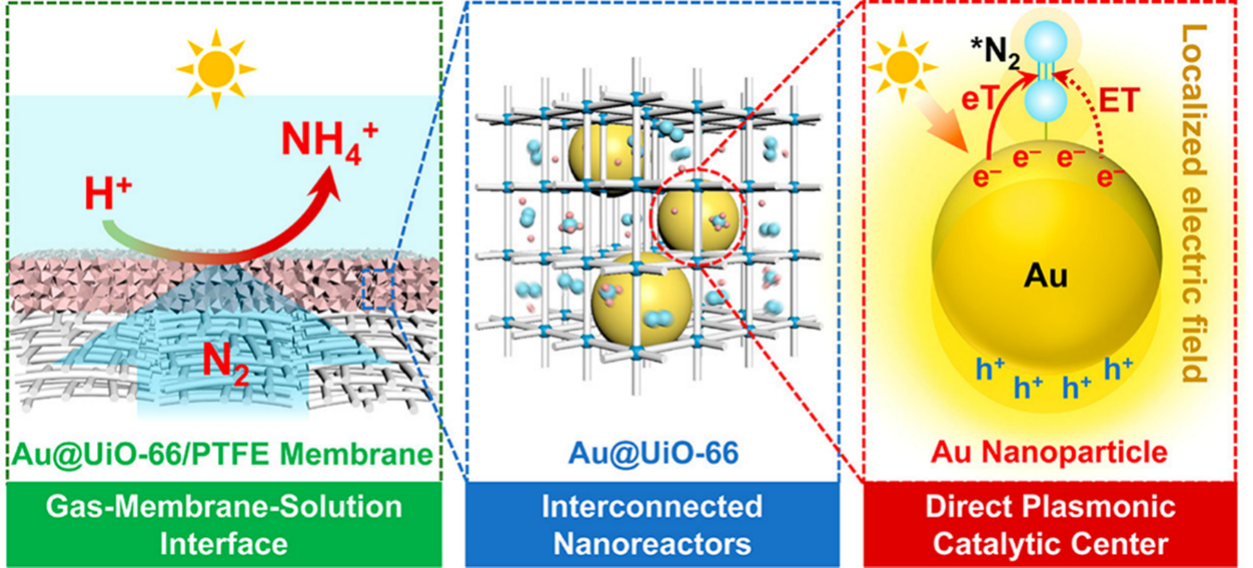
Schematic illustration for direct NRR on AuNPs encapsulated in UiO-66 matrix. Reproduced with permission from ref (31). Copyright ACS 2021.

In addition to acting as a single photocatalyst for NRR, MOFs have also been adopted within composites in combination with other active components. For example, the Zn-based MOF, TMU-5, and perovskite, KNbO_3_, were co-precipitated to form the composite photocatalyst KNbO_3_@TMU-5, which shows a higher rate of formation for NH_3_ (39.9 μmol·h^–1^·g^–1^) than KNbO_3_ alone (20.5 μmol·h^–1^·g^–1^) under similar reaction conditions.^29^ In this case, TMU-5 was shown to modify the charge-transfer and recombination behavior of KNbO_3_, and the improved catalytic activity was due to a combination of higher surface area, higher electron–hole separation efficiency, and higher electron density at the Nb sites within the composite. However, in another case, the photocatalyst, Bi_4_O_5_Br_2_@ZIF-8, incorporating the MOF component ZIF-8, was unable to participate in the charge-transfer process due to its wider bandgap and the mismatch of CB/VB (conduction band/valence band) potentials to those of Bi_4_O_5_Br_2_.^30^ Nonetheless, Bi_4_O_5_Br_2_@ZIF-8 still shows a rate of formation for NH_3_ (327 μmol·h^–1^·g^–1^) that is 3.6 times higher than that with pure Bi_4_O_5_Br_2_. This enhancement can be attributed to the triphasic reaction system that is created at the interface of the hydrophobic ZIF-8 and hydrophilic Bi_4_O_5_Br_2_, allowing direct supply of N_2_ from the gaseous phase. Here, the hydrophobicity of ZIF-8 was exploited to enhance the mass transfer of N_2_. The role of the MOF was also exemplified in another three-component composite catalyst, namely Au@UiO-66/PTFE (PTFE = polytetrafluoroethylene).^31^ In this system, gold nanoparticles (AuNPs) were encapsulated within a UiO-66 particle/membrane, and this achieved a rate of formation for NH_3_ as high as 810 μmol g_Au_^–1^ h^–1^. In this composite catalyst, AuNPs serve as the photosensitizer, co-catalyst, and plasmonic promoter for activation and reduction of N_2_. The highly dispersed AuNPs generate hot electrons upon visible light irradiation, and these transfer to adsorbed N_2_ molecules to catalyze their conversion to NH_3_ (Figure 4). Localized surface plasmon resonance (LSPR)-mediated energy transfer and localized electric field polarization effects are promoted by the AuNPs, thus greatly reducing the activation barrier and facilitating the activation of adsorbed N_2_ molecules. UiO-66 provides high surface area (∼1000 m^2^ g^–1^) for the diffusion of N_2_ molecules and (hydrated) protons to the plasmonic AuNPs, and also facilitates the dispersion and stabilization of AuNPs in achieving their optical–catalytic properties. While most heterogeneous photocatalytic NRR reactions are carried out with powders of catalyst suspended and gaseous N_2_ bubbled into the solution, here the MOF/PTFE permeable membrane was successfully fabricated to enable the direct feed of N_2_ gas to one side of the membrane coupled to the introduction of water/protons to the other side. This design overcomes the limited solubility and sluggish diffusion of N_2_ in aqueous solutions and improves the efficiency and applicability of the overall photocatalytic processes.

## 3 MOF-Assisted Electrochemical NRR

**Figure 5:**
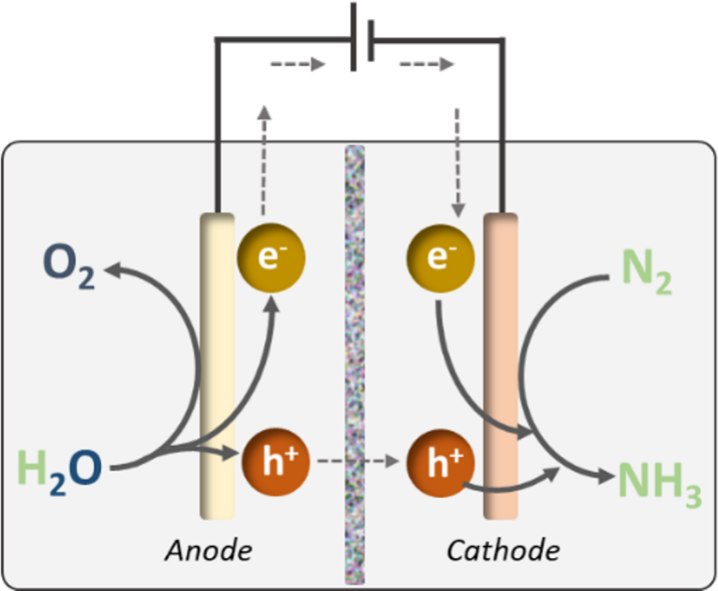
Schematic of the general electrochemical setup to produce NH_3_ from N_2_.

Electrochemical NRR is considered a green method because mature technologies exist to generate renewable electricity from wind, sun, and marine power.^32−34^ Since these energy sources tend to be intermittent and rely heavily on location and conditions, it is particularly appealing to develop a strategy which converts electricity into storable chemical energy such as NH_3_. Early examples of electrochemical NRR date back to the 1960s,^35,36^ and over the past 20 years electrochemical synthesis of NH_3_ over heterogeneous catalysts under ambient conditions has aroused significant interest. The fundamental target for the chemical reaction involves protons, formed by splitting of water at the anode, transferring to the cathode to combine with N_2_ and electrons to form NH_3_ (Figure 5). This seemingly simple process can involve complicated mass and energy transfer between components in multi-phase aggregates, and the configuration of the electrochemical cell, nature of the electrodes, and choice of the electrolyte all play crucial roles in the performance of the overall catalytic system. The addition of protons to N_2_ and its intermediates at the cathode is often recognized as the rate-determining step of this conversion, and the search for highly active cathodic catalysts to facilitate this addition represents a major focus. Various types of materials have been explored, including noble metals, transition metals, metal complexes, and conducting polymers, and the respective progress in this area has been summarized in two recent reviews.^37,38^ Here we pay closer attention to examples where MOFs and MOF-derived materials have been investigated as cathodic catalysts for electrochemical production of NH_3_ and identify future directions of research.

The most common method for preparing MOF-based cathodes is to disperse the MOF or MOF composite in a solution containing a conductive organic polymer, typically Nafion. This suspension is then loaded onto a commercial electrode, such as carbon paper or copper foam, followed by drying. A variety of units have been used in the literature for reporting the rate of formation of NH_3_. These have been converted here to μg_NH_3__·h^–1^·mg_cat._^–1^ wherever possible for the sake of uniformity and clarity. However, it is important to note that the reported rates of formation of NH_3_ reflect the performance of the entire electrochemical system, of which the cathode catalyst is only one of the albeit key components. In addition, differences and variations in conditions of catalysis such as temperature, electrolyte, and applied potentials make it difficult to compare directly and rank the catalytic activity of the MOFs/MOF composites between different studies based only on the rate of formation of NH_3_. Additionally, in many recent studies, MOFs have not only been used solely or directly as the cathodic catalyst, but also are converted to carbon-based materials or used as additives or coatings for functional electrodes to promote the overall production of NH_3_ within electrochemical systems.

### 3.1 MOFs as Electrocatalysts for NRR

Some benchmark MOFs have been screened as electrocatalysts for NRR. For example, MIL-100(Fe), ZIF-67(Co), and HKUST-1(Cu) were deposited onto carbon paper and served as cathodic catalysts.^39^ Of these, the highest observed rate of formation for NH_3_ was 22.3 μg_NH_3__·h^–1^·mg_cat._^–1^ with a Faradaic efficiency (FE) of 1.43% achieved using MIL-100(Fe) at 1.2 V vs Ag/AgCl and 90 °C with N_2_ and water as reactants. This performance is comparable to that of many other electrocatalytic systems for NH_3_ production using both metal and non-metal catalysts, and validates the activity of MOFs in the electrochemical reduction of N_2_.^40^ Based upon this work, two other Fe-based systems incorporating coordinatively unsaturated Fe(III) sites, namely MIL-88B-Fe and NH_2_-MIL-88B-Fe, were investigated.^41^ An optimal rate of formation for NH_3_ of 14.75 μg_NH_3__·h^–1^·mg_cat._^–1^ with a FE of 5.66% at −0.45 V versus RHE (reversible hydrogen electrode) was achieved using NH_2_-MIL-88B-Fe, higher than that with MIL-88B-Fe (4.38 μg_NH_3__·h^–1^·mg_cat._^–1^; FE of 5.59%). Although both of these systems show slower rates of formation of NH_3_ than MIL-100(Fe), it is worth noting that the study using MIL-100(Fe) was conducted at 90 °C as opposed to room temperature for MIL-88B-Fe and NH_2_-MIL-88B-Fe. Temperature can greatly affect the catalytic performance of the MIL-100(Fe) system. For example, on decreasing the temperature from 90 to 50 °C, the formation of NH_3_ is observed to reduce from 22.3 to 8.4 μg_NH_3__·h^–1^·mg_cat._^–1^_._

**Figure 6:**
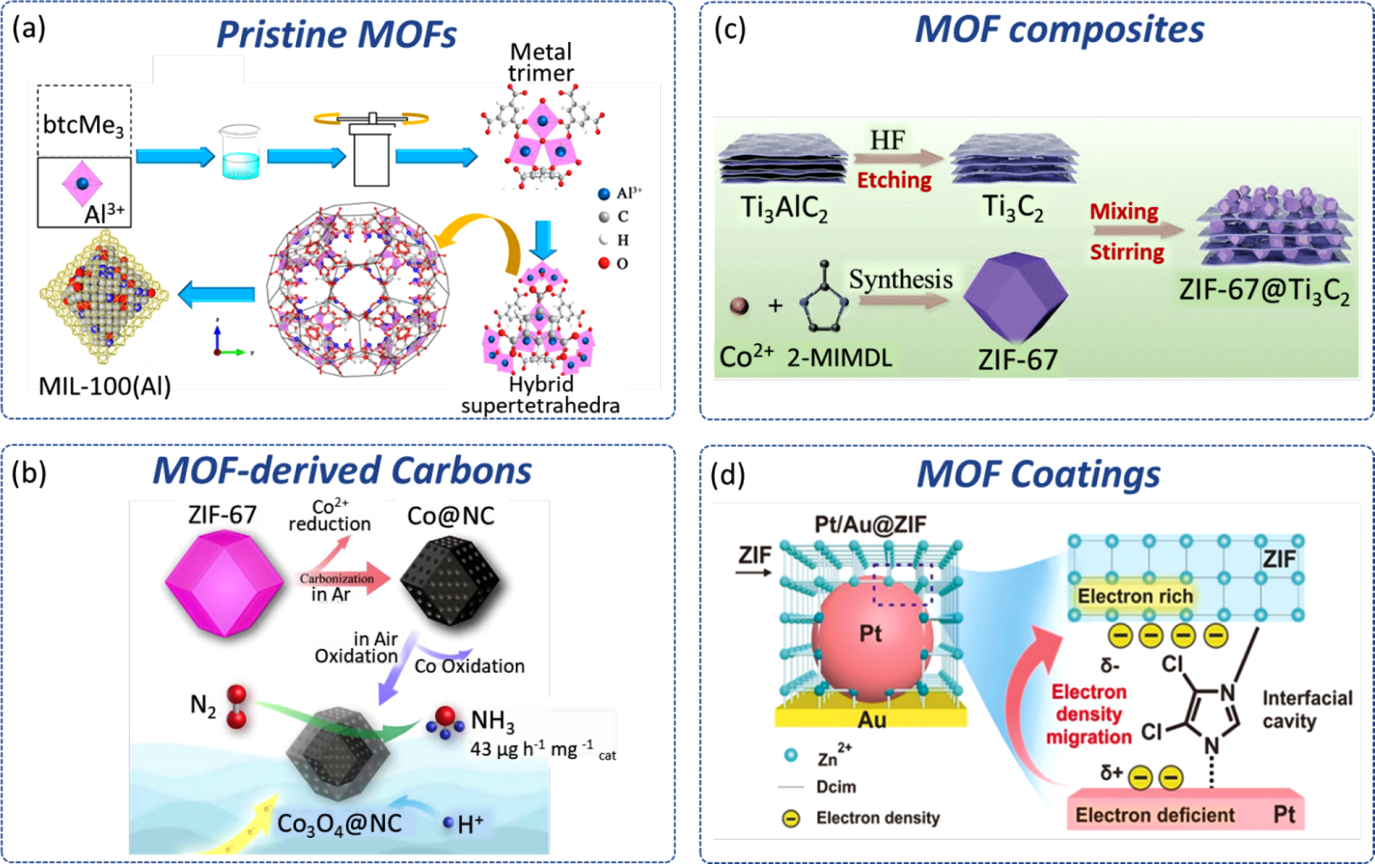
Schematics showing the preparation of representative examples of MOF-derived catalysts for electrochemical NRR. (a) Synthetic procedure of MIL-100(Al). Reproduced with permission from ref (42). Copyright ACS 2020. (b) Annealing process for the production of Co_3_O_4_@NC. Reproduced with permission from ref (54). Copyright ACS 2019. (c) Fabrication of ZIF-67@Ti_3_C_2_ composite. Reproduced with permission from ref (63). Copyright RSC 2021. (d) Surface electronic structure of the ZIF-modified Pt/Au catalyst. Reproduced with permission from ref (65). Copyright Wiley-VCH 2020.

In addition to transition metals, main group elements such as Bi, Al, and B are also highly active for the NRR due to their strong binding capacity for N_2_. MIL-100(Al), which has the same topology as MIL-100(Fe), was investigated for NNR (Figure 6a).^42^ An optimal rate of formation for NH_3_ of 10.6 μg_NH_3__·h^–1^·mg_cat._^–1^ and FE of 22.6% was reported for MIL-100(Al), higher than for MIL-53(Al) (∼2.1 μg_NH_3__·h^–1^·mg_cat._^–1^) and defect-MIL-100(Al) (∼2.0 μg_NH_3__·h^–1^·mg_cat_^–1^). While the introduction of defects to crystalline frameworks has been demonstrated to be an effective way of enhancing catalytic activity of MOF-based electrocatalysts,^43^ it is interesting to note that in this case defect-MIL-100(Al) shows lower NNR activity than defect-free MIL-100(Al).

Thus far, examples of using pristine MOFs for electrochemical NRR are still extremely limited, and these reported MOFs are composed of fairly simple organic ligands. They are known to possess low conductivities which likely significantly constrains their electrocatalytic activity. The conductive Co_3_HHTP_2_, constructed using the hexahydroxytriphenylene (HHTP) linker, was investigated for electrochemical NNR.^44^ The optimal rate of formation of NH_3_ was 22.1 μg_NH_3__·h^–1^·mg_cat_^–1^ with a FE of 3.34% at −0.40 V vs RHE in 0.5 M LiClO_4_. Considering the intrinsic electron conductivity is up to 11.50 S cm^–1^ for Co_3_HHTP_2_,^45^ and only 8.09 × 10^–5^ S cm^–1^ for MIL-100(Fe),^46^ the increase in the rate of formation of NH_3_ using Co_3_HHTP_2_ suggests that electron conductivity within the catalyst is only one of many factors contributing to optimal NH_3_ formation for a given electrochemical NNR system.

### 3.2 MOF-Derived Carbon Electrocatalysts for NRR

Rather than using pristine MOFs as catalysts, an alternative strategy is to use MOFs as sacrificial templates to generate porous carbon materials. This has been proven to be advantageous in many electrochemical reactions with observed improved conductivity and higher selectivity toward desired products compared with the pristine MOF.^47,48^ For example, ZIF-67(Co) has been used as a precursor to fabricate Co@N-doped carbon electrocatalysts for the reduction of N_2_.^49−51^ Upon annealing at high temperature (400–900 °C) for several hours under N_2_, the cubic ZIF-67(Co) transforms from crystals to porous carbons containing a high content of pyridinic N, pyrrolic N, graphic N, and Co-N_*x*_ moieties in the structure, which could all benefit or play a role in the adsorption and activation of N_2_. The rate of formation of NH_3_ for these Co@N-doped carbons under optimal conditions show dramatic differences of 5.1,^49^ 19.2,^51^ and 80.0^50^ μg_NH_3__·h^–1^·mg_cat._^–1^ between different studies. Assuming that the ZIF-67 precursors used in each study is of the same crystal structure and phase purity, the subsequent procedures for transforming it into the electrochemical catalysts include annealing, washing, dispersion, and deposition, and they appear to greatly affect the observed catalytic performance. The precise role of the carbon materials remains unclear because none of the studies on ZIF-67 derived Co@N-doped carbons compared their performances with pristine ZIF-67(Co) under identical conditions; one study claimed enhanced activity for Co@N-doped carbons over ZIF-67(Co).^49^ The reported Co@N-doped carbons did, however, show consistently much higher value of FE (10.1%, 11.5%, and 21.8%) than pristine ZIF-67(Co) (0.93%). This is most likely due to the improved conductivity of the carbon materials over the crystalline starting MOF material.

Annealing is also a versatile method for the inclusion of other elements into the resultant carbon materials. For example, because metal oxides^52^ and metal sulfides^53^ are recognized as important types of electrochemical catalyst for NNR, Co_3_O_4_@NC and CoS_2_@NC were prepared by introducing oxygen or sulfur during the annealing of ZIF-67. Co_3_O_4_@NC with core–shell structures can be obtained by controlling the time of annealing, and the obtained catalysts possess rich oxygen vacancies and show rates of formation for NH_3_ of 42.6 μg_NH_3__·h^–1^·mg_cat._^–1^ and FE of 8.5%, better than those obtained over Co_3_O_4_ nanoparticles and commercial Co_3_O_4_ (Figure 6b).^54^ In CoS_2_@NC, CoS_2_ nanoparticles are uniformly embedded in N-doped carbon, and this CoS_2_@NC catalyst exhibits a rate of formation for NH_3_ of 17.5 μg_NH_3__·h^–1^·mg_cat._^–1^ and FE of 4.6% under optimal conditions, higher than that with CoS_2_.^55^ For both Co_3_O_4_@NC and CoS_2_@NC, better catalytic performances were observed compared with the bare oxide and sulfide nanoparticles with nanoparticles confined within the ZIF-67-derived N-doped carbons. Several factors could potentially contribute to this improvement, including stronger binding to N_2_, enriched active vacant sites, smaller particle size, and facilitated mass and electron transfer. Significant additional comprehensive studies on a wider range of systems are required to reveal explicitly the dominant role of the carbon matrix in these composite catalysts.

In addition to ZIF-67(Co), two other MOFs, namely MIL-88B(V)^56^ and MIL-125(Ti),^57^ have also been used as templates for fabricating metal oxides doped carbon electrocatalysts. Upon annealing in Ar at 700 °C, MIL-88B(V) was converted to V_2_O_3_/C with its shuttle-like morphology retained. An optimal rate of formation for NH_3_ of 12.3 μg_NH_3__·h^–1^·mg_cat._^–1^ and FE of 7.28% were achieved with V_2_O_3_/C deposited on carbon paper, higher than with MIL-88B(V) or V_2_O_3_ deposited on carbon paper. As mentioned in the previous section, MIL-125(Ti) can be functionalized with light-responsive ligand and function as photocatalysts for NRR.^22^ It can also serve as a precursor for fabrication of carbon-doped and oxygen-deficient titanium oxide/carbon as electrocatalysts for NRR. The obtained C-Ti_*x*_O_*y*_/C affords rich (O−)Ti–C bonds and oxygen vacancies within its nanostructure, and a rate of formation for NH_3_ of 14.8 μg_NH_3__·h^–1^·mg_cat._^–1^ and FE of 17.8% under optimal conditions are observed. It is worth noting that the annealing temperature has a prominent effect on the structure, composition, porosity, and NRR activity of C-Ti_*x*_O_*y*_/C catalysts. In comparison with the commercial TiO_2_ and TiC catalysts and the carbon-doped rutile/TiC catalyst, it has been suggested that the (O−)Ti–C bonds in C-Ti_*x*_O_*y*_/C act as the active sites for fixation and activation of N_2_. This is further supported by DFT calculations on reduction of N_2_ over C-doped TiO_2_(110) surface (C-Ti_*x*_O_*y*_) and over non-doped oxygen-vacancy-enriched TiO_2_ (OVs-TiO_2_). The C-Ti_*x*_O_*y*_ moiety exhibits a low energy barrier for the formation of the potential rate-determining N–H* intermediates.

In addition to the metal nodes that constitute the framework structure, additional metal centers can be introduced into MOFs before converting the composite into carbon materials. For example, ZIF-8 has been used to synthesize single atoms of Ru incorporated into N-doped carbon (Ru SAs/N-C), which has been used as a cathodic catalyst for electrochemical NRR.^58^ Thus, upon annealing of Ru-loaded ZIF-8, the Zn centers of ZIF-8 evaporate leaving Ru centers bound to N sites as single atoms within Ru SAs/N-C. This material exhibits a higher NH_3_ productivity and FE (121 μg_NH_3__·h^–1^·mg_cat._^–1^ and 29.6%, respectively) than Ru nanoparticles incorporated into N–C (62 μg_NH_3__·h^–1^·mg_cat._^–1^ and 14.1%, respectively) under optimal conditions. The enhanced performance of Ru SAs/N-C is attributed to the presence of the atomically dispersed Ru sites within the ZIF-8-derived N–C matrix, affording improved binding to and activation of N_2_ compared to bulk Ru nanoparticles. Similarly, (Fe-N/C)-based catalysts can be prepared by annealing Fe-doped ZIF-carbon nanotube (CNT) templates, and the resultant hierarchical porous architecture affords a high electrochemically active surface area that is positively charged and shows weak ferromagnetism and strong chemisorption of N_2_.^59^ The highest rate of formation for NH_3_ was achieved using Fe-N/C-CNTs, 34.8 μg_NH_3__·h^–1^·mg_cat._^–1^ with a corresponding FE of 9.28% at −0.2 V vs RHE. This is higher than with bare CNTs (0.45 μg_NH_3__·h^–1^·mg_cat._^–1^) and N/C-CNTs (7.71 μg_NH_3__·h^–1^·mg_cat._^–1^). The DFT-calculated free-energy diagram revealed that the most favorable pathway for electroreduction of N_2_ occurred at Fe–N_2_ active sites, with the hydrogenation of the adsorbed N_2_ molecule to [N_2_H] species assigned as the potential rate-limiting step. More recently, bio-inspired bimetallic carbon materials were obtained using PMo_12_@MIL-100(Fe)@PVP as the precursor *via* an *in situ* one-step hydrothermal sulfuration method.^60^ By altering the annealing temperature and time, both Fe_1.89_Mo_4.11_O_7_/FeS_2_@C and FeMoO_4_/FeS_2_@C electrocatalysts can be formed. The former exhibits a rate of formation for NH_3_ of 105 μg_NH_3__·h^–1^·mg_cat._^–1^ and FE of 54.7%, outperforming the latter (51.0 μg_NH_3__·h^–1^·mg_cat._^–1^ and 43.9%). Studies of the mechanism based upon DFT calculations revealed that N_2_ was activated *via* formation of moderate Mo–N interaction with the Mo(II) site of the catalyst. This facilitates the adsorption of *N–NH and removal of *NH_2_ species along with the reaction pathway. Here, the role of the MOF is beyond its intrinsic structural properties, and the MOF appears primarily to assist the fabrication of complex composite catalysts by integrating multiple components. The wide dispersion of the metal sites across the framework, the inclusion of additional polyoxometalates, and the chemical affinity to organic polymers all facilitate the formation of the carbon-based catalysts for NRR.

MOFs can also be used to fabricate metal-free carbon catalysts. For example, when ZIF-8(Zn) is used as a precursor, Zn can be removed completely during annealing at high temperatures (1100 °C) due to its relatively low melting point (907 °C). The resultant nanoporous carbon catalyst is thus metal-free.^61^ During annealing, the well-dispersed ZIF-8 nanocrystals fuse together to form highly disordered N-doped and defect-rich carbon structures. By tuning and optimizing the doping concentration of nitrogen and the degree of graphitization, a rate of formation for NH_3_ of 73.2 μg_NH_3__·h^–1^·mg_cat._^–1^ and an FE of 10.2% at −0.3 V vs RHE can be achieved at room temperature and ambient pressure in 0.1 M KOH electrolyte. While pristine ZIF-8 shows a much lower rate of formation for NH_3_ (<4.3 μg_NH_3__·h^–1^·mg_cat._^–1^) and loss of N content during the electrocatalysis, nitrogen-doped carbon (N@C) exhibits excellent stability over an 18 h continuous test with constant production rates. Interestingly, while previous studies indicate that noble or transition metal species within the carbon-based catalysts promote the catalytic activity for NRR,^58−60^ some adverse effects of the doped Fe within the ZIF-derived N@C was observed due to active sites within the N@C being blocked by Fe. This was observed to facilitate the competing hydrogen evolution reaction (HER). The various observations for different catalytic systems clearly indicate a chemical complexity around the role of doped metal centers in these systems, depending on their electronic nature, coordination environment within the carbon matrix, and their activity toward side reactions. Future efforts are required to unravel the precise local structure of doped metal sites within these carbon matrices using *operando* investigations to monitor the change of coordination environment as a function of applied potential and upon binding of N_2_.

### 3.3 MOF Composites as Electrocatalysts for NRR

While converting MOFs to carbon material appears to be an effective methodology, the annealing process almost always require high temperatures which makes the production of the catalyst an energy-intensive process. Another proposed strategy to improve the electron conductivity of MOFs is to combine them with highly conductive nanostructures to form composite materials. For example, carbon nanotubes (CNT) and N-doped CNTs (NCNT) have been inserted into UiO-66, BIT-58, CAU-17, and MIL-101, to form CNT/NCNT@MOFs composites where the CNT and NCNT serve as the catalytic center and provide electron conduction pathways. The hydrophobic MOF facilitates local N_2_ enrichment and desorption of the NH_3_ product.^62^ With these composite catalysts, an optimal rate of formation for NH_3_ ranging from 3.81 to 13.3 μg_NH_3__·h^–1^·mg_cat._^–1^ can be achieved; this is generally higher than those achieved with bare CNT and NCNT (1.54 and 3.16 μg_NH_3__·h^–1^·mg_cat._^–1^, respectively). Meanwhile, the optimal FEs for these CNT/NCNT@MOFs composites, ranging between 12.4 and 37.3%, are also much higher than those observed for bare CNT and NCNT (1.77% and 1.87%, respectively). This suggests that the synergic effects of confined electrocatalysis, selective chemical diffusion, and electrical transport have been amplified through the construction of composite electrocatalysts. Another example involves supporting ZIF-67(Co) on Ti_3_C_2_ (MXene) to form ZIF-67@Ti_3_C_2_*viain situ* growth (Figure 6c). This aims to combine the high porosity and large active surface area of ZIF-67 with the ultra-high conductivity of Ti_3_C_2_.^63^ The resultant ZIF-67@Ti_3_C_2_ composite demonstrates higher NRR activity with rates of formation for NH_3_ of 112 μg_NH_3__·h^–1^·mg_cat._^–1^ and FE of 20.2% compared with bare ZIF-67 (27.6 μg_NH_3__·h^–1^·mg_cat._^–1^) and Ti_3_C_2_ (47.6 μg_NH_3__·h^–1^·mg_cat._^–1^). However, due to the lack of in-depth investigation of the catalytic mechanism, the molecular details of the activity of this multi-component catalyst for electrochemical NRR remain unclear. But, this simple procedure for producing MOF-based composites will surely inspire further exploitation of various combinations of MOFs and conducting materials to catalyst discovery. Insights from *in silico* studies or machine learning will benefit greatly such experimental exploration.

For electrochemical NRR in aqueous solutions, HER from water is a significant competitive reaction that limits the FE of NRR. To unblock this bottleneck, non-aqueous electrochemical systems have been developed, for example using dry THF as solvent, EtOH as proton source, LiCF_3_SO_3_ as electrolyte, and noble metal (Ag/Au and Pt/Au) coated with ZIF-71 as electrocatalysts.^64,65^ In the case of Ag/Au coated with ZIF-71, the coating mainly serves as a hydrophobic layer to repel trace amounts of water in THF from reaching the catalyst. The ZIF-71 coating on Pt/Au can also lower the electronic d-band center of Pt nanoparticles by withdrawing electrons from Pt to ZIF-71 *via* Pt–N_ZIF_ interactions (Figure 6d). This ZIF-71-induced reduction in surface electron density of Pt was shown to weaken Pt–H formation and simultaneously create electron deficient affinity sites for adsorption of N_2_. This led to a high rate of formation for NH_3_ of 161 μg_NH_3__·h^–1^·mg_cat._^–1^ and an increased FE (44%) of NRR by >44-fold compared with the system in the absence of ZIF-71. Along with these examples where MOFs are coated on the surface of relatively large NPs (Ag NPs: 119 ± 4 nm, Pt NPs: 60 nm), a recent example demonstrated the capability of a Zn-MOF to host much smaller Au NPs (1.9 ± 0.4 nm) for the electrocatalytic conversion of N_2_ in aqueous electrolytes.^66^ A similar strategy of coating the catalyst with a hydrophobic layer was applied to suppress the competing HER reaction, and the formation rate of NH_3_ was boosted to 49.5 μg_NH_3__·h^–1^·mg_cat._^–1^ with FE of 60.9% by coating the catalyst with a layer of hydrophobic organosilicone.

## 4 MOFs for NH_3_ Capture and Storage

The technologies for the photochemical and electrochemical production of NH_3_ are appealing from an environmental perspective and are denoted as “Gen3-Green NH_3_”.^67^ These technologies are expected to enter the market at scale toward the end of the 2020s and to contribute significantly to global NH_3_ production thereafter. However, one main practical issue in scaling up such processes is separating the product from the reaction mixtures. In general, the rate of production of NH_3_ from N_2_ is low, and since the flow rate of N_2_ used in the reaction process is relatively high, the exhaust gas of the reactor comprises a mixture of N_2_ containing low concentrations of NH_3_, typically less than 100 ppm. In laboratory research, the NH_3_-containing gas stream is often passed through an acidic solution for analysis, but for any practical applications where anhydrous NH_3_ is the desired product, follow-on separation and enrichment of NH_3_ from the gas steam are required. In this case, the established cryogenic separation of NH_3_ in the Haber–Bosch process is energy-inefficient because of the relatively low concentrations of NH_3_ in the gas stream, and thus separation technologies based on membrane and sorption become important. As porous sorbents with diverse structural features, MOFs have been investigated extensively for separating gas mixtures, and exceptional selectivity and capacity using their tunable porosity and rich functionality have been observed.^68,69^ The adsorption of NH_3_ in MOFs^70−73^ and MOF-based composite^74^ materials has recently been discussed in a few recent reviews.

MOFs are often unstable to NH_3_, and this remains a major hurdle in applying them for the capture and storage of NH_3_. Effective strategies for the construction of stable MOFs have been proposed and developed, for example, strengthening the metal–ligand bond, using kinetically inert metal, increasing the connectivity of the framework components, and creating steric shielding of metal–ligand bonds.^75^ Here, we highlight a few MOFs that display high stability and can be applied for the capture of low concentrations of NH_3_ from gas streams. MIL-53, NH_2_-MIL-53, MIL-100, and MIL-101 have been studied for NH_3_ adsorption. Although only moderate capacities of 4.4, 5.4, 8.0, and 10.0 mmol g^–1^ were achieved, respectively, at 298 K and 1 bar, it is encouraging that all four MOFs retain their capacities over five cycles of adsorption/desorption.^76^ To promote the adsorption capacity, Lewis acidic open metal sites are often recognized as a desirable feature of MOFs for providing binding interaction to substrates, but common materials that incorporate open metal sites, such as HKUST-1^77^ and MOF-74,^78^ show very limited stability upon exposure to NH_3_, especially under humid conditions. A series of materials [M(NA)_2_] (M = Zn, Co, Cu, Cd; NA^−^ = nicotinate) with open metal sites have been synthesized using environmentally friendly methods.^79^ These complexes are flexible and retain adsorption capacity for NH_3_ over three cycles of isothermal adsorption/desorption up to 1 bar. However, the isotherms exhibit a broad hysteresis loop, and complete regeneration of the materials can only be achieved by applying dynamic vacuum at elevated temperatures. Recently, a series of triazolate MOFs with a high density of open metal sites, [M_2_Cl_2_BTDD]^80^ (H_2_BTDD = bis(1*H*-1,2,3-triazolo[4,5-*b*],[4,5-*i*])dibenzo[1,4]dioxin) and [M_2_Cl_2_BBTA]^81^ (H_2_BBTA = 1*H*,5*H*-benzo(1,2-*d*:4,5-*d*′)bistriazole), exhibited much improved stability for adsorption of NH_3_ due to the strong coordinate bond between the donor triazolate linkers and the metal centers. For [M_2_Cl_2_BTDD], isostructural Mn, Co, and Ni materials maintain a high uptake of NH_3_ of 15.5, 12.0, and 12.0 mmol g^–1^, respectively, at STP (standard temperature and pressure) over three cycles. The density of open metal sites within the framework can be increased further by using the shortened ligand H_2_BTTA, and the resultant MOFs, [M_2_Cl_2_BBTA] (M = Co, Ni, and Cu) are of smaller pore diameter and show higher NH_3_ uptake of 18.0, 14.7, and 19.8 mmol g^–1^, respectively, at 298 K and 1 bar. However, the increased uptakes were achieved at the expense of stability, with only [Ni_2_Cl_2_BBTA] retaining its crystallinity upon pore filling with NH_3_. [M_2_(dobpdc)] (M = Mg, Mn, Co, Ni, Zn; dobpdc^4–^ = 4,4′-dioxidobiphenyl-3,3′-dicarboxylate) possess open metal sites and are constructed from tetradentate ligand with various divalent metal cations.^82^ Of these isostructural MOFs, [Mg_2_(dobpdc)] exhibits exceptional adsorption capacity values of 23.9 mmol g^–1^ at 1 bar and 8.25 mmol g^–1^ at 0.57 mbar. This material showed sufficient stability to retain its adsorption capacity over five consecutive cycles of breakthrough experiments with the feed stream of 1000 ppm of NH_3_ under wet conditions (80% relative humidity). Most recently, direct observation of binding of NH_3_ on unique, low-coordinate Cu(II) sites in a series of robust UiO-66 materials has been reported.^83^ While all three MOFs (UiO-66-defect, UiO-66-Cu^I^, and UiO-66-Cu^II^) exhibit similar surface areas (1111–1135 m^2^ g^–1^), decoration of the defect −OH sites in UiO-66-defect with Cu(II) sites results in a 43% enhancement of the isothermal uptake of NH_3_ in UiO-66-Cu^II^ (11.8 and 16.9 mmol g^–1^, respectively, at 273 K and 1.0 bar) and a 100% enhancement of dynamic adsorption of NH_3_ (2.07 and 4.15 mmol g^–1^, respectively, at 630 ppm and 298 K). *In situ* neutron powder diffraction, inelastic neutron scattering, electron paramagnetic resonance, solid-state nuclear magnetic resonance, and infrared spectroscopy, coupled with modeling, reveal the critical role of the near-linearly coordinated Cu(II) sites in binding NH_3_, representing the first example of structural elucidation of NH_3_ binding in MOFs containing open metal sites. The enhanced NH_3_ uptake of UiO-66-Cu^II^, therefore, originates from the strong [Cu(II)···NH_3_] interaction coupled with a reversible change of the near-linear coordination geometry of the Cu(II) site. Importantly, the high uptakes of NH_3_ in these UiO-66 materials can be fully retained after 15 cycles of adsorption/desorption.

MOFs incorporating coordinatively unsaturated metal centers are usually more vulnerable to collapse than those with metal centers that are fully saturated, especially in the presence of strong Lewis basic guest molecules such as NH_3_ and H_2_O. In this sense, ligand functionality has been recognized as a desirable strategy to introduce active sites onto the pore-wall for the selective capture of NH_3_. For example, MFM-300(Al) features a 3D open framework consisting of hydroxyl-decorated [AlO_4_(μ_2_-OH)_2_]_∞_ chains linked by organic linkers in a “wine-rack” mode. This MOF demonstrated structural stability with a series of toxic and corrosive gases, including NH_3_, for over 4 years.^84^ It also retained its uptake of NH_3_ (13.9 mmol g^–1^ at 273 K and 1 bar) over 50 cycles of adsorption/desorption. This excellent stability originates from the strong coordinate bond between the Al(III) and carboxylate ligands, and a mechanism of adsorption based upon reversible H/D site exchange between the adsorbent and adsorbate.^85^ Isostructural MFM-300(M) [M = V(IV), Fe(III), V(III), and Cr(III)] show high NH_3_ uptakes of 17.3, 16.1, 15.6, and 14.0 mmol g^–1^ at 273 K and 1 bar, respectively, which can also retain over at least 20 adsorption/desorption cycles.^86^ The incorporation and use of hydroxyl groups for NH_3_ adsorption have also been exemplified by an Al-based MOF with a porphyrin ligand (Al-PMOF), where isothermal uptake of 7.67 mmol g^–1^ at 1 bar and 298 K can be retained for two cycles.^87^ In addition, the high stability of Al-PMOF enables loading of HCl and HCOOH, and the resultant Al-PMOF-HCl and Al-PMOF-HCOOH species can achieve 7.9 and 5.5 wt% breakthrough capacities, respectively, for NH_3_ under 80% relative humidity.^87^ More recently, a dual-functionalized MOF with free carboxylic acid and hydroxyl groups, MFM-303(Al), has been reported to show reversible adsorption of NH_3_ up to 9.9 mmol g^–1^ at 273 K and 1 bar, and the unique pore environment results in an exceptional packing density for NH_3_ at 293 K (0.801 g cm^–3^) compared with that of solid NH_3_ at 193 K (0.817 g cm^–3^).^88^

The above reports provide valuable information on the feasibility of using MOFs to capture low concentrations of NH_3_ from gas mixtures. However, none of these studies have been targeted at the application of enrichment of NH_3_ from the gaseous product mixtures derived from electrochemical NRR. Therefore, more rigorous studies on the stability in humid environments and cyclic regeneration are required before MOFs can be directed to practical capture of NH_3_. A particular target would be the successful enrichment of NH_3_ from NRR mixtures, and using dynamic adsorption experiments with a N_2_ stream containing diluted NH_3_ (<500 ppm) at high relative humidity (>90%) at a total flow rate no lower than 20 mL min^–1^. After adsorption reaches saturation, desorption through pressure or temperature swing will be required to quantify the amount and purity of NH_3_ upon regeneration of the sorbent; this will indicate the online productivity of NH_3_ of the studied electrochemical NRR process. Finally, reuse of the sorbent for multiple cycles of capture/release process is required to demonstrate its long-term stability.

Compared with H_2_, there is a high level of maturity in many aspects of the infrastructure for NH_3_ storage and transport because of its widespread use as a feedstock for fertilizers. As an indication of scale, it is common to see NH_3_ storage tanks with capacity over thousands of liters, and there are those that can store NH_3_ of up to 50,000 tonnes. Unless MOFs with high storage capacity can be produced at scale with competing cost to demonstrate their technical feasibility, it is unlikely that MOFs would replace the current infrastructure for large-scale static storage of NH_3_. However, there are still potential needs for new storage technologies especially with the expanded end-use of NH_3_. There are several power technologies that are compatible with NH_3_ as a transport fuel, such as reaction with O_2_ from the air in a fuel cell or burning within internal combustion engines and gas turbines. It is particularly suitable to transport modes where large amounts of energy are required for extended periods of time and where batteries or direct electrical connection are not practical or cost-effective, such as heavy good vehicles, trains, aviation, and long-distance shipping. In these cases, sorption-based storage technologies display clear advantages for safe storage and spillage elimination. As shown by some of the above examples, exceptional packing density of NH_3_ had been achieved within functionalized MOFs owing to their high porosity and active sites for binding NH_3_.^88^ Furthermore, the application of MOFs as efficient NH_3_ sorbents will also help to promote the social acceptance of NH_3_ as a large-scale fuel and energy carrier.

## 5 Outlook

### 5.1 Photocatalytic NRR over MOF-Based Catalysts

Although MOFs currently account for a very small proportion of photocatalysts investigated for NRR, each of the above examples demonstrates the potential of MOFs and related materials and composites. Enhanced response to visible light can be achieved *via* functionalization of the bridging organic ligand, and redox activity can be controlled by manipulating the coordination environment at the metal center. The overall catalytic activity can be explored and optimized by utilizing the porosity and selective capture and binding of substrates within a controlled environment with targeted defect and active sites. Theoretical screening can offer useful insights to the design of MOF-based photocatalysts for NRR. In a recent study, a hypothetical library of MOFs was screened based upon consideration of structure, adsorption, environmental, cost, and optical properties.^89^ Zn-BTC (BTC^3–^ = benzene-1,3,5-tricarboxylate) incorporating phenyl functionality satisfied all the preliminary screening criteria and is predicted to be superior to the experimentally studied MIL-125(Ti).^89^ As adsorption of reactant is almost always a prerequisite to its activation and catalytic conversion, MOFs that show strong binding to N_2_ molecules are particularly worth exploring. For example, calculations based on quantum mechanical computations have predicted that V-MOF-74 with open V(II) sites could show high enthalpy of adsorption for N_2_ due to back-bonding interactions from electron-rich metal centers to the π* orbitals of N_2_.^90^ However, the synthesis of crystalline V-MOF-74 is yet to be achieved. In another example, MIL-100(Cr) with unsaturated Cr(III) centers was reported for its unusual ability to capture N_2_ over CH_4_ and O_2_. Formation of single quasi-linear N–N–Cr^3+^ adducts within the pores was observed, and the fact that this MOF can thermodynamically capture N_2_ over O_2_ may make it even more appealing when air is used as the source of N_2_.^91^

It is evident that significant more work is required to achieve the full potential of MOFs for photochemical NRR. Future directions for developing MOF-based photocatalysts could focus on two aspects. (i) Creating metal sites that favor the binding of nitrogen. This is one of the greatest strengths of MOFs compared with other types of photocatalysts, such as dense-phase metal oxides and carbon-based materials. Through the coordination to versatile organic ligands, not only the electronic configuration and stereochemistry of the metal centers can be manipulated, but also the confined space around the metal node can be finely tuned to bind and trap N_2_. (ii) Boosting the performance of composite catalysts by utilizing porosity, hydrophobicity, and electron-transfer properties of MOFs. For state-of-the-art photocatalysts for NRR, such as g-C_3_N_4_-based catalysts,^92,93^ careful design and systematic investigations to rationalize the function of MOFs are required. As composite catalysts are inevitably more complex in structure and function, characterization of their interfacial structure and reaction pathways within these integrated systems is required.

### 5.2 Electrochemical NRR over MOF-Based Catalysts

A transition from state-of-the-art Haber–Bosch process to new technologies to produce NH_3_ using renewable resources and energy is essential to its implementation as a sustainable fuel for global use in the future.^67^ There is significant commercial interests in the production of renewable NH_3_ and for the development of technologies to allow the extraction of H_2_ to power fuel-cell vehicles, a process that creates more than 10 times the value when NH_3_ is used as fertilizer.^94^ Compared to the enormous the numbers of MOFs now known (>100,000) and the availability of MOF-based electrochemical catalysts being applied to O_2_ reduction and evolution,^95^ CO_2_ reduction,^96^ and HER reactions,^97^ the electrochemical NRR using MOF materials is still very much in its infancy. However, even with the above limited examples, MOFs have already been shown to exhibit versatility and advantageous features for electrochemical NRR. They have been applied as a sole catalyst, as a precursor for generating carbon-based catalysts, as active components within composite catalysts, and as modifying coatings or hosting scaffold for noble metal catalysts. The above exploratory research clearly signals the great potential of MOF and related hybrid materials for the electrochemical NRR and as catalysts for a range of other important transformations.

Future directions in this area will likely focus on five aspects:

(i) *Design and screening MOF-based electrocatalysts for NRR using theoretical calculations.* Although the discovery and optimization of catalysts are often approached using a trial-and-error approach, which is time and labor consuming, the rational screening of catalysts using computational methods could potentially accelerate this process. For electrochemical NNR, screening of various types of catalysts, such as transition-metal surfaces, nanoclusters, single atoms, transition-metal nitrides, carbides, and oxides, has been performed,^37^ but up to now, computational studies on MOF-based electrocatalysts for NRR remain scarce.^98^ Functionalization and choice of the bridging ligand, judicious choice of metal centers, and tuning of porosity and coordination environment all have pronounced effects on the activity of MOF catalysts. Thus, the construction and optimization of the structure of MOF catalysts at an atomic level will represent a foundation to achieve better performance.

(ii) *Improving the integration of the design of the electrochemical device for NRR.* Compared with the vessels used for most thermally driven reactions, electrochemical cells often contain more components and have more parameters to tune in order to optimize the performance of the reactions. Especially for NRR, where both gaseous and liquid reactants are involved, the cell configuration, choice of the support electrolyte, and morphology of the electrodes will all play significant roles in mass and energy transfer of the reaction.

(iii) *Refining the preparation of MOF-based electrodes*. Currently, most MOF-based cathodes are prepared *via* dispersion of MOF particles in a chosen solvent containing Nafion. This involves dropping known amounts of the suspension onto the conducting surface of an electrode (*e.g.*, carbon paper), followed by washing and drying of the electrode. This procedure is facile but offers limited control over the morphology and microstructure of the catalysts. For other important electrochemical conversions (e.g., CO_2_ reduction, O_2_ reduction, O_2_ evolution, H_2_ oxidation, and H_2_ evolution), various strategies have been explored to fabricate MOF-based electrocatalysts (*e.g.*, interfacial synthesis, template-assisted construction, chemical vapor deposition),^99^ and these techniques are also applicable to the construction of MOF-based catalysts for electrochemical NRR. These different methods in combination will allow the fine-tuning of important parameters of the active electrode, such as its chemical composition and morphology, which determine its hydrophobicity, permeability, and overall catalytic activity for NRR.^100^

(iv) *Clarifying the role of MOFs in catalytic NRR*. For electrochemical NRR, the MOF, MOF-composite, or MOF-derived catalyst is immersed in a complex chemical and electrochemical environment. It not only contains the reactants (N_2_, H_2_O, and H^+^) and the possible products (NH_3_, NH^4+^, and N_2_H_4_), but also always contains counterions within the aqueous electrolyte, such as OH^–^, Cl^–^, ClO_4_^–^, Na^+^, and K^+^. During the catalytic reaction with a voltage applied, the surface of the MOF catalyst is also charged to establish an electrical double layer (EDL), and the local concentration of species within the EDL region is significantly different from that of the bulk solution. The structural stability of MOFs and the derived catalytically active species under these conditions requires careful investigation and analysis in order to demonstrate the real performance of the catalysts and to provide insights into the reaction mechanism. It is worth noting that the reconstruction or even destruction of the structure of the pristine MOFs (also termed as “structural evolution”) during electrochemical NRR may not necessarily lead to a decrease in the catalytic performance of the system. In some cases, MOFs can be regarded as precatalysts for the formation of highly active species that catalyze electrochemical conversions.^101−105^

(v) *Improving the electrical conductivity of the catalysts.* In electrochemical NRR, the catalyst is often involved in both intrinsic (between the catalyst and the substrates) and extrinsic (between the catalyst and the electrode) electron-transfer processes, and high electrical conductivity is a desirable feature. However, MOFs are generally recognized as insulators and are of low electrical conductivity due to their structures based on coordinate bonding between the insulating organic ligands and metal nodes. As discussed previously, various strategies have been developed for improving the conductivity of MOF-based catalysts for NRR, such as carbonization of MOFs and inclusion of conductive species. Considering the many other choices of carbon sources available (*e.g.*, biomass and petroleum chemicals), MOF-derived carbon materials will need to show much stronger catalytic performance to demonstrate economic feasibility for such processes. On the other hand, direct design of electrically conductive MOFs has been investigated for diverse applications such as electrochemical conversion and energy storage in recent years, and the progress in these areas has been summarized.^106,107^ With successful examples of conductive MOFs being applied to oxygen reduction reactions,^108,109^ oxygen evolution reactions,^110,111^ and hydrogen evolution reactions,^112,113^ future research to exploit conductive MOFs for electrochemical NRR appears to be a very promising approach.

Although the efficiency and productivity of photo- and electrocatalysis for NRR are still far from meeting industrial or commercial needs, the structures and versatile properties of MOFs do imply that there is ample room for improvements. The development of an effective catalyst requires understanding of the underlying catalytic mechanism which is essential in informing the design of future catalysts. The crystalline nature and rich functionality of MOFs could benefit such *operando* investigation using advanced crystallographic, scattering, and spectroscopic techniques. This remains an almost unexplored area to date for NRR. Finally, each photo-/electrocatalytic NRR reaction is a complex system involving multi-phasic reactants, complicated mass and energy transfer, light irradiation, and/or electrical conductivity. The intrinsic activity of the catalyst can only be fully realized taking a systems approach when all components of the catalytic system work synergistically to reach optimal conditions.
